# Hepatic Mitochondrial Dysfunction and Immune Response in a Murine Model of Peanut Allergy

**DOI:** 10.3390/nu10060744

**Published:** 2018-06-08

**Authors:** Giovanna Trinchese, Lorella Paparo, Rosita Aitoro, Carmela Fierro, Michela Varchetta, Rita Nocerino, Maria Pina Mollica, Roberto Berni Canani

**Affiliations:** 1 Department of Biology, University of Naples “Federico II”, 80126 Naples, Italy; giovanna.trinchese@unina.it (G.T.); mariapia.mollica@unina.it (M.P.M.); 2 Department of Translational Medical Science—Pediatric Section, University of Naples “Federico II”, 80131 Naples, Italy; paparolorella@gmail.com (L.P.); aitoro.rosita@gmail.com (R.A.); carmelafierro0@gmail.com (C.F.); mic.varchetta@studenti.unina.it (M.V.); ritanocerino@alice.it (R.N.); 3 European Laboratory for the Investigation of Food-Induced Diseases (ELFID), University of Naples “Federico II”, 80131 Naples, Italy; 4 CEINGE Advanced Biotechnologies, University of Naples “Federico II”, 80131 Naples, Italy; 5 Task Force on Microbiome Studies, University of Naples “Federico II”, 80131 Naples, Italy

**Keywords:** food allergy, mitochondrial function, oxidative stress, Th2 cytokines

## Abstract

Background: Evidence suggests a relevant role for liver and mitochondrial dysfunction in allergic disease. However, the role of hepatic mitochondrial function in food allergy is largely unknown. We aimed to investigate hepatic mitochondrial dysfunction in a murine model of peanut allergy. Methods: Three-week-old C3H/HeOuJ mice were sensitized by the oral route with peanut-extract (PNT). We investigated: 1. the occurrence of effective sensitization to PNT by analysing acute allergic skin response, anaphylactic symptoms score, body temperature, serum mucosal mast cell protease-1 (mMCP-1) and anti-PNT immunoglobulin E (IgE) levels; 2. hepatic involvement by analysing interleukin (IL)-4, IL-5, IL-13, IL-10 and IFN-γ mRNA expression; 3. hepatic mitochondrial oxidation rates and efficiency by polarography, and hydrogen peroxide (H_2_O_2_) yield, aconitase and superoxide dysmutase activities by spectrophotometry. Results: Sensitization to PNT was demonstrated by acute allergic skin response, anaphylactic symptoms score, body temperature decrease, serum mMCP-1 and anti-peanut IgE levels. Liver involvement was demonstrated by a significant increase of hepatic Th2 cytokines (IL-4, IL-5 and IL-13) mRNA expression. Mitochondrial dysfunction was demonstrated by lower state 3 respiration rate in the presence of succinate, decreased fatty acid oxidation in the presence of palmitoyl-carnitine, increased yield of ROS proven by the inactivation of aconitase enzyme and higher H_2_O_2_ mitochondrial release. Conclusions: We provide evidence of hepatic mitochondrial dysfunction in a murine model of peanut allergy. These data could open the way to the identification of new mitochondrial targets for innovative preventive and therapeutic strategies against food allergy.

## 1. Introduction

Food allergy (FA), defined as an adverse immune response to food proteins, is a major public health issue in Western countries due to its increasing prevalence and severity as well as the negative impact on quality of life and medical care costs [1]. Peanut allergy (PA) is one of the most common types of FA [2]. The prevalence of PA among children in Western countries has doubled in the past 10 years, reaching rates of 1.4–3.0% [3]. In contrast to other FA, such as cow milk allergy, the majority of PA cases persist throughout life and are often associated with life-threatening symptoms [4]. Thus, the investigation of new targets for effective preventive and therapeutic strategies is highly advocated. Compelling evidence has been recently accumulated that mammals’ mitochondria have multiple critical roles in immunity, and that in addition to being the powerhouse of the cell, they also represent the powerhouse of immunity [5]. In this light, increasing evidence strongly suggests a relevant role for mitochondrial dysfunction, and consequent excessive generation of reactive oxygen species (ROS) in allergy [6,7,8,9,10,11,12,13,14]. Mitochondrial dysfunction and elevated ROS have been reported in atopic dermatitis, allergic rhinitis and asthma [9,13,15,16]. The involvement of liver in FA is emerging [17]. An increased risk for FA in patients affected by severe liver damage has been demonstrated [18,19]. Studies in a murine model suggest that the liver could act as a source of CD4^+^ T cells and could play an important role in the IgE response to dietary antigens [20]. Despite the central role played by the liver in the maintenance of immune-metabolic homeostasis being well accepted, the involvement of hepatic mitochondrial function in FA is largely undefined. We aimed to investigate hepatic mitochondrial dysfunction in an animal model of PA.

## 2. Materials and Methods 

### 2.1. Animals

For all experiments, three-week-old female C3H/HeOuJ mice (Charles River Laboratories—Calco, Lecco, Italy) were used, as previously reported (21). Mice were housed in the animal facility under a 12L:12D lighting cycle, 20–24 °C range of ambient temperature and 40–70% relative humidity. The mice were acclimated to their environment for 1 week before experiments and were divided into two groups (*n* = 6). All procedures involving the animals were carried out in accordance with the Institutional Guidelines and complied with the Italian D.L. no.116 of 27 January 1992 of the Italian Ministry of Health and associated guidelines in the European Communities Council Directive of 24 November 1986 (86/609/ECC). Experiments were approved by the Institutional Committee on the Ethics of Animal Experiments (CSV) of the University of Naples “Federico II” and by the Minister of Health (protocol no. 2012-0024683).

### 2.2. Materials

All chemicals used were analytical grade and were purchased from Sigma (St. Louis, MO, USA), unless otherwise specified.

### 2.3. Sensitization Protocol

**Figure 1:**
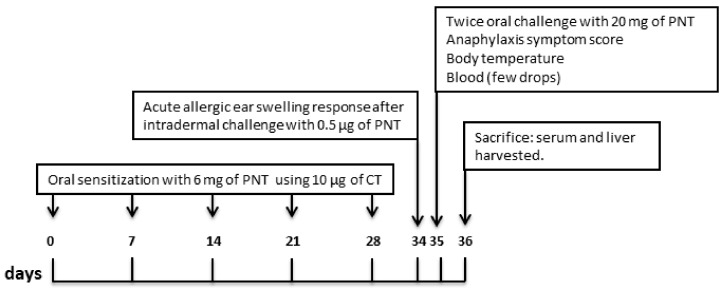
Schematic overview of the experimental design. Three-week-old female C3H/HeOuJ mice (*n* = 6 per group) were sensitized orally every 7 days for 4 weeks using a blunt needle with peanut extract (PNT) + cholera toxin (CT) as adjuvant. Controls mice receive CT only. On day 34, mice received intradermal injection of PNT in the ear pinnae acute allergic skin response was measured. After 24 h, mice were challenged by gavage with PNT and anaphylaxis score and body temperature were determined. On the next day mice were sacrificed, liver and blood samples were collected.

The experimental design is reported in Figure 1. As previously described [21], mice were sensitized orally using a blunt needle on days 0, 7, 14, 21, and 28 with 6 mg of purified PNT (kindly provided by Prof. C. Nagler) [22] mixed with 10 µg of cholera toxin (CT) (Sigma-Aldrich, Steinheim, Germany) as adjuvant [23] in Tris buffer as a vehicle. We used purified PNT prepared from roasted, unsalted peanuts by a modification of van Wijk et al., which omitted high-speed centrifugation at 10,000× *g* [24]. Control groups received CT only, in Tris buffer as a vehicle. One week after the final sensitization, acute allergic skin response was assessed. The next day, rectal temperature was measured. Mice were then challenged twice with 20 mg of PNT delivered by gavage 30 min apart, and after 1 h, anaphylaxis score was assessed, and rectal temperature was measured again. On the subsequent day, mice were sacrificed, blood samples were collected, and livers were aseptically excised and processed. Liver samples not immediately used for mitochondrial preparation were frozen and stored at −80 °C for subsequent determinations. The experiment was repeated twice. 

### 2.4. Acute Allergic Skin Response, Anaphylaxis Symptom Score, Body Temperature and mMCP-1 Serum Level

Acute allergic skin response was evaluated according to a previously described procedure [25]. Ear thickness was measured in duplicate using a digital micrometre (Mitutoyo, Lainate, Italy) 1 h after intradermal injection of 0.5 μg of PNT in the ear pinnae by an investigator blind to the study group assignment. The ear swelling was calculated by correcting the allergen-induced ear thickness with the basal ear thickness. The delta ear swelling was shown in μm units. Hypersensitivity symptoms were scored by an investigator blind to the study group assignment 1 h after the second oral challenge: 0 = no symptom; 1 = scratching and rubbing around the nose and head; 2 = reduced activity; 3 = activity after prodding and puffiness around the eyes and mouth; 4 = no activity after prodding, laboured respiration, and cyanosis around the mouth and the tail; and 5 = death [26]. Rectal body temperature was measured before and every 5 min for 1 h after second challenge by rodent thermometer (Bioseb, Valbonne, France). To assess mast cell degranulation, blood was collected by submandibular bleeding methods to determinate mMCP-1 serum levels 1 h after second challenge according to the manufacturer’s protocol using a commercially available ELISA kit (Thermo Fischer Scientific, Waltham, MA, USA). For mMCP-1 determination, each sample was tested in triplicate. 

### 2.5. Serum Anti-Peanuts Immunoglobulin E

Blood samples obtained by intracardiac puncture from mice were collected into serum separator tubes. The serum portion was separated by centrifugation at 10,000× *g* for 5 min at 20 °C. Serum samples were then aliquoted into Eppendorf tubes and stored at −20 °C until analysis. Anti-peanut serum immunoglobulin IgE was detected by ELISA, as previously described [22]. 96 multiwell plates (Sigma-Aldrich, Steinheim, Germany) were coated with 100 μg/mL of PNT in 0.1 mol/L Na-bicarbonate/carbonate coating buffer (pH 9.6). PNT-IgE standards were prepared from the serum of sensitized mice by affinity purification on a PNT-conjugated CNBr-Sepharose column (GE Healthcare, Little Chalfont, United Kingdom), as previously described [22]. After overnight incubation at 4 °C, plates were washed 3 times with 150 μL of PBS plus 0.05% Tween-20 (PBS-T) and blocked with 100 μL of 2% BSA in PBS-T for 2 h at 37 °C. Subsequently, the plates were washed 3 times and 100 μL of serially undiluted serum samples were added to the wells and incubated at 37 °C for 90 min. Plates were then washed 3 times and 100 μL of Biotin Rat-anti-mouse IgE (R35-118, BD Biosciences, Milano, Italy) were added to each well. The plates were incubated at 37 °C for 2 h and washed 5 times. Then, 100 μL of horseradish peroxidase-conjugated streptavidin (Streptavidin HRP—554066, BD Biosciences, Milano, Italy) was added to each well and the plates were again incubated at 37 °C for another 60 min and washed 3 times. Then, 100 μL of TMB (3, 3′, 5, 5′tetramethyldiaminebenzidine) was added to each well followed by a 15 min incubation step to allow for the development of colorimetric reactions. Absorbance was read at a wavelength of 450 nm in a microplate reader. Each sample was tested in triplicate. 

### 2.6. Cytokines Gene Expression Analysis by Quantitative Real Time PCR

Total RNA was isolated from portions of mice liver by solubilisation in Trizol (Invitrogen Life Technologies, Carlsbad, CA, USA) and quantified using the Nanodrop 2000c spectrophotometer (Thermo Scientific, Waltham, MA, USA). For cDNA synthesis, 1 μg total RNA was transcribed using the High Capacity cDNA Reverse Transcription kit (Applied Biosystems, Foster City, CA, USA) according to the manufacturer’s instructions. Quantitative Real Time PCR (qRT-PCR) analysis of IL-4, IL-5, IL-13, IL-10 and IFN-γ was performed using TaqMan specific probes (Applied Biosystems, Grand Island, NY, USA) (Table 1). Each sample was run in triplicate at 95 °C for 30 s followed by 40 cycles of 95 °C for 10 s and 60 °C for 30 s, using a Light Cycler 7900HT (Applied Biosystems). Data analysis was performed using the comparative threshold cycle (CT) method. Quantitative gene expression was calculated with the comparative CT method and normalized against the CT of the Glucuronidase (GUS) messenger reference gene, as previously used by Gong et al. [27].

### 2.7. Preparation of Isolated Mitochondria

After removal, another portion of the liver from the same mice was finely minced and washed in a medium containing 220 mM-mannitol, 70 mM sucrose, 20 mM -*N*′-(2-hydroxyethyl)piperazine-*N*-2-ethanesulfonic acid (HEPES) (pH 7.4), 1 mM-EDTA, and 0.1% (*w*/*v*) fatty-acid-free bovine serum albumin (BSA). Tissue fragments were homogenised with medium (1:4, *w*/*v*) in a Potter Elvehjem homogeniser (Heidolph, Kelheim, Germany) set at 500 g/min (4 strokes/min). The homogenate was centrifuged at 1000 g_av_ for 10 min and the resulting supernatant fraction was again centrifuged at 3000 g_av_ for 10 min. The mitochondrial pellet was washed twice and finally re-suspended in a medium containing 80 mM-KCl, 50 mM-HEPES (pH 7.0), 5 mM KH_2_PO_4_, and 0.1% (*w*/*v*) fatty-acid-free BSA. The protein content of the mitochondrial suspension was determined by the Hartree [28] method using BSA as the protein standard. 

### 2.8. Oxidative Capacity

Mitochondrial O_2_ consumption was estimated by a Clark type electrode (Yellow Springs Instruments, Yellow Springs, OH, USA) maintained in a water-jacketed chamber at 30 °C. Hepatic mitochondria (0.5 mg protein) were incubated in a medium containing 80 mM KCl, 50 mM HEPES, 1 mM EGTA, 5 mM KH_2_PO_4_ (pH 7.0), and 0.1% (*w*/*v*) fatty-acid-free BSA, as previously described [29]. The substrates used for liver respiration were 10 mM succinate + 3.75 μM rotenone or 40 μM palmitoyl-carnitine + 2.5 mM-malate for the determination of fatty acid oxidation rate. State 3 measurements were performed in the presence of 0.6 mM ADP. State 4 respiration was measured after ADP exhaustion. The ratio between state 3 and 4, called the respiratory control ratio, was calculated according to Estabrook [30]. The addition of ADP after the substrate to the mitochondrial incubation allows to the ATP synthase to function and to electron transport chain to accelerate (‘state 3_ADP_’). When the ATP/ADP ratio approaches equilibrium proton re-entry through the ATP synthase stops and respiration slows (‘state 4’) [31]. In control experiments, we assessed the purity of mitochondrial preparation by checking a possible contamination by other ATPase-containing membranes <10%, whereas the quality of mitochondrial preparation was assessed by adding cytochrome c (3 nmol/mg protein) and evaluating an enhancement in state 3 respiration rate ≤10%, as previously indicated [32,33]. The degree of coupling was determined in the liver by applying equation by Cairns et al. [34]: degree of coupling = [1 − (Jo)sh/(Jo)unc]^1/2^ where (Jo)sh represents the oxygen consumption rate in the presence of oligomycin that inhibits ATP synthase, and (Jo)unc is the uncoupled rate of oxygen consumption induced by carbonyl-cyanide-*p*-trifluoromethoxyphenylhydrazone (FCCP), which dissipates the transmitochondrial proton gradient. (Jo)sh and (Jo)unc were measured as above using succinate (10 mmol/L) rotenone (3.75 µmol/L) in the presence of oligomycin (2 µg/mL) or FCCP (1 µmol/L), respectively.

### 2.9. Determination of Superoxide Dismutase (SOD) and Aconitase Enzymatic Activities and H_2_O_2_ Release

SOD specific activity was measured in a medium containing 0.1 mM EDTA, 2 mM KCN, 50 mM KH_2_PO_4_, pH 7.8, 20 mM cytochrome c, 5 mM xanthyne, and 0.01 U of xanthyne oxidase. Determinations were carried out spectrophotometrically (550 nm) at 25 °C, by monitoring the decrease in the reduction rate of cytochrome c by superoxide radicals, generated by the xanthine–xanthine oxidase system. One unit of SOD activity is defined as the concentration of enzyme that inhibits cytochrome c reduction by 50% in the presence of xanthine + xanthine oxidase [35]. Aconitase activity was obtained by solubilizing mitochondria (40–60 μg) in 1% Triton X-100 and enzymatic activity was measured in a medium containing 30 mmol/l sodium citrate, 0.6 mmol/L MnCl_2_, 0.2 mmol/L NADP, 50 mmol/L Tris-HCl, pH 7.4, and 2 units of isocitrate dehydrogenase. The formation of NADPH was analysed spectrophotometrically (340 nm) at 25 °C. Aconitase activity measured is equal to the active aconitase (basal level). Aconitase inhibited by ROS in vivo was reactivated by incubating mitochondrial extracts in a medium containing 50 mM dithiothreitol, 0.2 mM Na_2_S, and 0.2 mM ferrous ammonium sulphate [36]. Mitochondrial H_2_O_2_ release was measured at 30 °C following the linear increase in fluorescence (excitation at 320 nm, emission at 400 nm) due to oxidation of homovanillic acid by H_2_O_2_ in the presence of horseradish peroxidase in a computer-controlled Jascofluorometer equipped with a thermostatically controlled cell-holder. Known concentrations of H_2_O_2_ were used to establish the standard concentration curve [37].

### 2.10. Statistical Analysis

The Kolmogorov-Smirnov test was used to determine whether variables were normally distributed. To evaluate the differences among continuous variables, the independent sample *t*-test was performed. Pearson’s correlation coefficient ‘r’ was used to evaluate the correlation between continuous variables. The level of significance for all statistical tests was 2-sided, *p* < 0.05. All analyses were conducted by a statistician, using SPSS version 19.0 for Windows (SPSS Inc., Chicago, IL, USA) and Graph Pad Prism 5.0. 

## 3. Results

### 3.1. Allergic Response and Liver Involvement in Peanut Extract-Sensitized Mice

**Figure 2:**
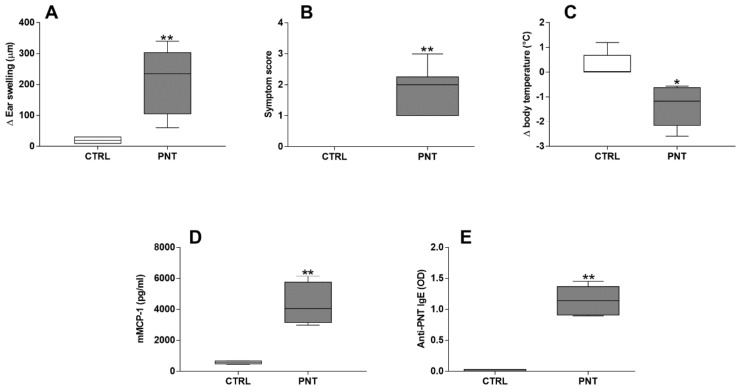
Proofs of effective sensitization to PNT. Acute allergic ear swelling response 1 h after intradermal injection of PNT in the ear pinnae (**A**), anaphylactic symptoms score (**B**), body temperature decrease (**C**), serum levels of mMCP-1 (**D**) and anti-peanut IgE (**E**). Data were analyzed with independent sample *t*-test. CTRL = control mice; PNT = mice sensitized with peanut extract. * vs. CTRL, *p* < 0.05; ** vs. CTRL, *p* < 0.0001. Data represent pooled values from two separate experiments.

**Figure 3:**
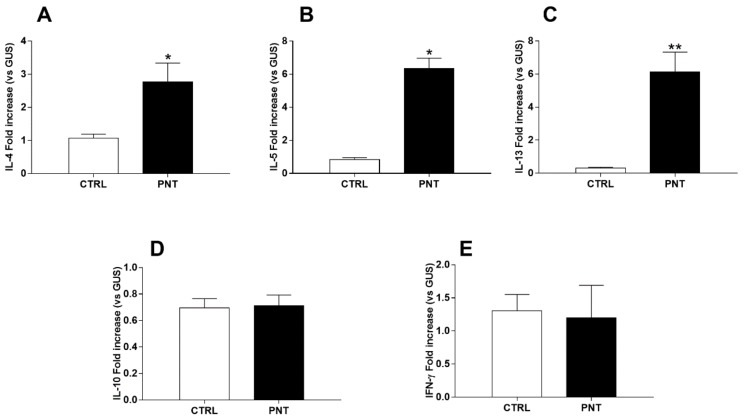
Proofs of hepatic involvement in Th2 response in mice sensitized with PNT: increase in IL-4 (**A**), IL-5 (**B**) and IL-13 (**C**) mRNA liver expression detected by Real Time PCR. No significant difference in IL-10 (**D**) and IFN-γ (**E**) mRNA liver expression was observed between two groups. Data are reported as means ± SEM. Data were analysed with independent sample *t*-test. CTRL = control mice; PNT = mice sensitized with peanut extract. * vs. CTRL, *p* < 0.05; ** vs. CTRL, *p* < 0.0001.

Effective sensitization to PNT was demonstrated by acute allergic skin response (213.5 ± 44.46 vs. 20 ± 3.65; *p* < 0.0001), anaphylactic symptoms score (1.8 ± 0.31 vs. 0 ± 0; *p* < 0.0001), body temperature decrease (−1.4 ± 0.33 vs. 0.3 ± 0.2; *p* < 0.05), serum mMCP-1 (4264 ± 484.3 vs. 569.6 ± 37.3; *p* < 0.0001) and anti-PNT IgE levels (1.146 ± 0.09 vs. 0.015 ± 0.006; *p* < 0.0001) (Figure 2). was suggested by the increase of hepatic IL-4 (2.77 ± 0.57 vs. 1.07 ± 0.012; *p* < 0.05), IL-5 (6.34 ± 0.63 vs. 0.83 ± 0.12; *p* < 0.05) and IL-13 (6.14 ± 1.19 vs. 0.31 ± 0.06; *p* < 0.0001) mRNA expression observed in mice sensitized with PNT Whereas, no modifications of IL-10 (0.71 ± 0.08 vs. 0.69 ± 0.07; *p* = 0.8698) and IFN-γ (1.19 ± 0.49 vs. 1.30 ± 0.25; *p* = 0.8556) mRNA liver expression were observed (Figure 3). 

### 3.2. Hepatic Mitochondrial Oxidative Capacity

**Figure 4:**
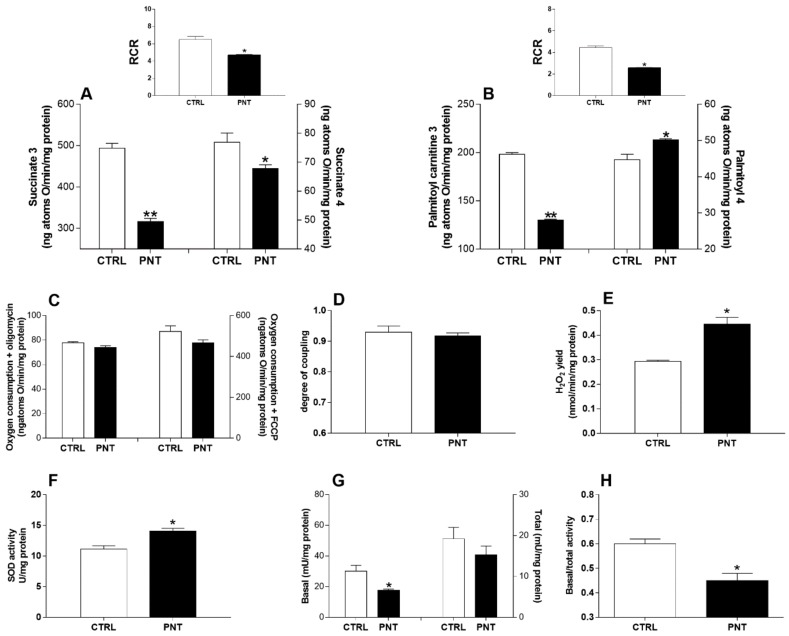
Evidence of hepatic mitochondrial dysfunction in mice sensitized by PNT. Hepatic mitochondrial state 3 and 4 respiration rates in the presence of succinate (**A**) or palmitoyl-carnitine (**B**) were evaluated, and the relative respiratory control ratios (RCRs) were shown (upper panels). Oxygen consumption in the presence of oligomycin or uncoupled by FCCP (**C**), and degree of coupling values (**D**) were also shown. The mitochondrial H_2_O_2_ yield (**E**), the SOD activity (**F**), the basal and total activity of the aconitase (**G**) and its ratio (**H**) were reported. Data are reported as means ± SEM from duplicate analyses. Data were analyzed with independent sample *t*-test. CTRL = control mice; PNT = mice sensitized with peanut extract. * vs. CTRL, *p* < 0.05; ** vs. CTRL, *p* < 0.0001.

Liver mitochondrial respiration rates, in the presence or absence of ADP (state 3 and state 4), evaluated using succinate (10 mM) as a substrate were significantly decreased in animals sensitized with PNT when compared to control mice (state 3, 316.3 ± 7.8 vs. 493.7 ± 11.8, *p* < 0.0001; state 4, 67.9 ± 1.2 vs. 76.9 ± 3.2, *p* < 0.05) (Figure 4A). Mitochondrial state 3, measured in the presence of palmitoyl-carnitine and malic acid as substrates, was significantly decreased in sensitized animals (state 3, 130.1 ± 1.0 vs. 198.3 ± 1.7, *p* < 0.0001), mitochondrial state 4 was significantly increase in mice sensitized with PNT when compared to control mice (50.2 ± 0.3 vs. 44.7 ± 1.5, *p* < 0.05) (Figure 4B). Respiration control ratio (RCR) values were indicative of high-quality mitochondrial preparations (Figure 4A,B upper panels) and confirmed the absence of necrotic injury as demonstrated by normal serum values of released hepatocellular transaminases and histological features of liver tissues checked at the end of each experiment (data not shown). Mitochondria generate ATP by oxidizing nutrients (glucose, FAs, and some amino acids), and the energy generated by the electron transport is utilized to phosphorylate ADP to ATP. Electron transport and ATP synthesis are tightly coupled, but some of the energy generated by electron transport is uncoupled from ATP synthesis. To test this mitochondrial efficiency, we measured oxygen consumption in presence of oligomycin and FCCP. State 4 respiration rate with oligomycin (73.8 ± 1.6 vs. 77.6 ± 1.1, *p* = 0.0001) and maximal FCCP-stimulated respiration (466.2 ± 15.3 vs. 522 ± 2.8, *p* = 0.1098) showed no variation in sensitized animals when compared to controls (Figure 4C). No difference in hepatic mitochondrial energetic efficiency, assessed as degree of coupling, was found between the two different groups of animals (0.917 ± 0.01 vs. 0.929 ± 0.02; *p* = 0.6032) (Figure 4D).

### 3.3. Hepatic Oxidative Stress

The sensitized animals showed an increase in liver mitochondrial ROS production as confirmed by a higher mitochondrial hydrogen peroxide (H_2_O_2_) yield (an indirect index of mitochondrial superoxide production) when compared to control mice (0.445 ± 0.028 vs. 0.293 ± 0.005; *p* < 0.05) (Figure 4E) despite a higher SOD activity (14.04 ± 0.49 vs. 11.15 ± 0.53; *p* < 0.05) (Figure 4F). Moreover, the sensitized animals showed the lower basal/total aconitase activity ratio (0.45 ± 0.03 vs. 0.6 ± 0.02; *p* < 0.05), a sensitive marker of oxidative stress (Figure 4G,H).

### 3.4. Correlation Analysis

Pearson correlation coefficients between hepatic mitochondrial function parameters, oxidative stress markers and interleukin mRNA expression levels were calculated. As shown in Table 2, a significant correlation between mitochondrial function markers, oxidation rates (succinate state 3 and palmitoyl-carnitine state 3), aconitase enzyme activity, and H_2_O_2_ release and anti-peanut IgE, IL-5 and IL-13 mRNA expression was observed. In particular, mitochondrial oxidation rates were negatively correlated with IL-4 mRNA liver expression; IL-5 and IL-13 mRNA liver expressions were negatively correlated with both mitochondrial oxidation rates and basal/total aconitase enzyme activity. Moreover, H_2_O_2_ production was positively correlated with Th2 cytokine mRNA liver expression. These results highlight the potential link between reduced liver mitochondrial activity and increased yield of ROS, which in turn correlated with anti-PNT IgE, IL-4, IL-5 and IL-13 expression.

## 4. Discussion

Our results provide evidence of liver mitochondrial dysfunction in FA. In sensitized mice, we found a lower state 3 respiration rate in the presence of succinate, a decreased fatty acid oxidation in the presence of palmitoyl-carnitine, an increased yield of ROS proven by the inactivation of aconitase enzyme and a higher H_2_O_2_ mitochondrial release despite the enhancement of SOD activity. All these features were not dependent on liver tissue damage and parallelized a significant increase of IL-4, IL-5 and IL-13 mRNA liver expression. 

Increasing evidence suggests a relevant role for mitochondrial dysfunction, and consequent excessive generation of ROS, in allergy [6,7,8,9,10,11,12,13,14]. A cross-talk between mitochondria and other cellular pathways in response to environmental factors through epigenetic mechanisms has been demonstrated in chronic immune-mediated conditions [38]. Our data are well in line with previous observations reporting mitochondrial involvement in many phases of an allergic response, including dendritic cell differentiation, Ag presentation, T cells activation, B cells proliferation and activation which are a prerequisite for IgE synthesis; release of mast cell mediators, including IL-4, produced by Th2 cell [39,40]. Mitochondrial dysfunction could precede allergic inflammation, as demonstrated in the airway epithelium of asthma animal model, where prior to antigen exposure, mitochondrial dysfunction exacerbates allergen-induced accumulation of eosinophils, mucin levels, and airway hyper responsiveness [9]. Accordingly, allergic response is enhanced in mice that have pre-existing defects in the mitochondrial electron transport chain [41], and polymorphisms in mitochondrial-encoded genes have been associated with increased IgE production and allergy [42]. More recently, mitochondrial Ca^2+^/calmodulin-dependent protein kinase II has emerged as key mediator of the molecular response in allergy, regulating eotaxin, IL-4, IL-5, IL-13, and eosinophilic inflammation [43]. Moreover, it has been demonstrated that antioxidants and n-3 fatty acids may exert a beneficial effect on allergic inflammation in humans [6,44,45,46,47,48] and that various nutritional components are able to influence metabolic homeostasis, by modulating mitochondrial function and ROS production [32,49,50]. Therefore, nutritional supplements can be evaluated to develop new therapeutic strategies for FA. All these data suggest a bidirectional cause-effect relationship between mitochondrial metabolic stress and pathogenesis of allergic inflammation [15,51]. 

Evidence suggests the importance of the liver as “regulatory system” where different immune and non-immune cell populations work together in preventing allergic response against gut-derived food allergens [52]. These immune cells coexist in a close symbiotic manner to support the hepatic metabolic functions [53]. In the liver, naïve T cells recirculating within the sinusoids make direct contact with sinusoidal cells, such as liver sinusoidal endothelial cells (LSECs) or Kupffer cells. Gut-derived food antigens are taken up by Kupffer cells, LSECs, and liver dendritic cells and presented to naïve T cells, leading to immune tolerance of both CD8^+^ T cells and CD4^+^ T cells [54]. Moreover, we cannot exclude that mitochondrial dysfunction in other organs and tissues could be associated to FA. This is a major limitation of the present study, together with the lack of data on possible different involvement of hepatic cell subpopulations in mitochondrial dysfunction and on other forms of FA induced by different antigens.

## 5. Conclusions

The results of this study provide the first evidence of mitochondrial dysfunction in a murine model of PA and add novel significant perspectives on the role played by hepatic mitochondria in FA. Further studies will be advocated to investigate the mechanistic connections between Th2 response and mitochondrial dysfunction that could elucidate the importance of mitochondria-targeted therapies as new age approach against FA. 

## Figures and Tables

**Table 1:** Probe TaqMan * details used for real-time PCR analysis.

Gene Symbol	RefSeq	Exon Boundary	Assay Location	Amplicon Length
IL5	NM_010558.1	2–3	219	62
IL4	NM_021283.2	2–3	241	79
IL13	NM_008355.3	1–2	211	56
IL-10	NM_010548.2	4–5	515	136
IFN-γ	NM_008337.3	3–4	469	100
GUSb	NM_010368.1	10–11	1657	71

* All TaqMan probe are inventoried and tested by Applied Biosystems manufacturing facility (QC).

**Table 2:** Correlation between mitochondrial function markers and Th2 response.

		Palmitoyl-Carnitine 3	H_2_O_2_	Basal/Total Aconitase	Anti-PNT IgE	IL-4	IL-5	IL-13
Succinate 3	*Pearson Correlation*	0.951 **	−0.864 **	0.778 **	−0.950 **	−0.588 *	−0.854 **	−0.742 **
	*Sig. (2-tailed)*	0.000	0.000	0.003	0.000	0.044	0.000	0.006
Palmitoyl-carnitine 3	*Pearson Correlation*	–	−0.873 **	0.786 **	−0.966 **	−0.683 *	−0.919 **	−0.832 **
	*Sig. (2-tailed)*		0.000	0.002	0.000	0.014	0.000	0.001
H_2_O_2_	*Pearson Correlation*		–	−0.848 **	0.0875 **	0.651 *	0.750 **	0.802 **
	*Sig. (2-tailed)*			0	0.000	0.022	0.005	0.002
Basal/total aconitase	*Pearson Correlation*			–	−0.875**	−0.376	−0.768 **	−0.642 *
	*Sig. (2-tailed)*				0.000	0.228	0.004	0.024
Anti-PNT IgE	*Pearson Correlation*				–	0.525	0.931 **	0.757 **
	*Sig. (2-tailed)*					0.079	0.000	0.004
IL-4	*Pearson Correlation*					–	0.600 *	0.849 **
	*Sig. (2-tailed)*						0.039	0.000
IL-5	*Pearson Correlation*						–	0.842 **
	*Sig. (2-tailed)*							0.001

* = Correlation is significant at the 0.05 level (2-tailed); ** = Correlation is significant at the 0.01 level (2-tailed).
